# Bouveret Syndrome Presenting as Duodenal Bulb Stenosis and Gastric Outlet Obstruction: A Case Report

**DOI:** 10.7759/cureus.104572

**Published:** 2026-03-02

**Authors:** Panagiotis G Doukas, Aadhithyaraman Santharaman, Alana B Barofsky, Marcella Pimpinelli, Arkady Broder

**Affiliations:** 1 Internal Medicine, Rutgers Robert Wood Johnson School of Medicine/Saint Peter’s University Hospital, New Brunswick, USA; 2 Medicine, Division of Gastroenterology and Hepatology, Rutgers Robert Wood Johnson School of Medicine/Saint Peter's University Hospital, New Brunswick, USA; 3 Medicine, Division of Gastroenterology and Hepatology, Rutgers Robert Wood Johnson School of Medicine/Saint Peter’s University Hospital, New Brunswick, USA

**Keywords:** bouveret's syndrome, cholecysto-duodenal fistula, gallstone disease (gsd), gastric outlet obstrction, urgent upper endoscopy

## Abstract

Bouveret syndrome, or gallstone obstruction of the duodenum, is a rare cause of gastric outlet obstruction, where gallstones migrate through a bilioenteric fistula and obstruct the pylorus or duodenum. Here, we present a case of a 61-year-old man who presented with intractable nausea and vomiting. Initial imaging, such as computed tomography of the abdomen and pelvis, showed gastric distention without an obvious obstructing lesion, and a subsequent upper endoscopy revealed a cholecystoduodenal fistula filled with gallstones, posterior to a stenotic region in the duodenal bulb. The calculi were spontaneously evacuated, the patient’s symptoms resolved, and the patient was referred for surgical management. This case highlights the diagnostic challenges of Bouveret syndrome, particularly in the setting of atypical imaging, and emphasizes the importance of timely endoscopic assessment in uncovering rare etiologies of gastric outlet obstruction.

## Introduction

Bouveret syndrome is a rare form of gastric outlet obstruction (GOO) caused by the impaction of a gallstone that has originated in the gallbladder and migrated through a biliary-enteric fistula into the duodenum or, less commonly, the pylorus [1]. It accounts for less than 3% of all gallstone ileus cases and under 1% of GOO [2,3]. The most frequently observed fistula is cholecystoduodenal, although cholecystocolonic has also been reported [4].

Risk factors include longstanding cholelithiasis, female sex, age over 60 years, and large gallstones (>2 cm) [3]. Symptoms are often nonspecific, including nausea and vomiting (up to 85%), abdominal pain (70%), weight loss, and abdominal distension [4]. Due to its rarity and vague presentation, diagnosis is frequently delayed, contributing to morbidity (60%) and mortality (12-30%) [3], with complications such as metabolic derangements and aspiration pneumonia.

In fewer than 50% of cases, a particular collection of signs, also known as Rigler's triad, may be observed, which includes pneumobilia, an ectopic gallstone, and signs of small bowel obstruction [4]. Computerized tomography (CT) scan offers >90% diagnostic accuracy [5], while endoscopy confirms diagnosis and may serve a therapeutic role, especially in high-risk surgical candidates. We present a case of Bouveret syndrome diagnosed via CT and esophagogastroduodenoscopy (EGD), emphasizing diagnostic and management challenges.

## Case presentation

**Figure 1:**
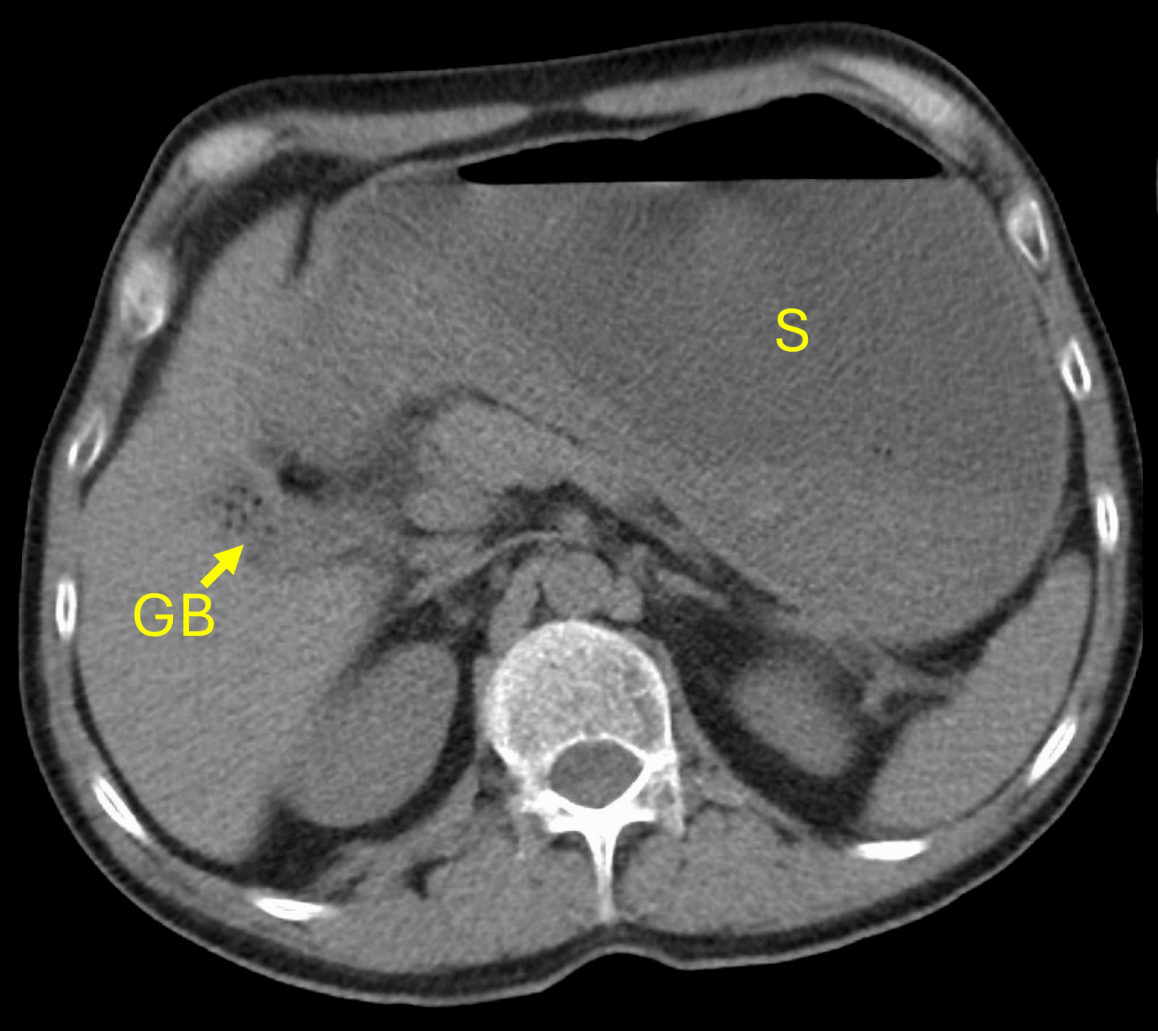
Imaging evaluation via CT scan of the abdomen Axial view of CT scan of the abdomen without oral contrast depicting the significantly distended stomach. S: stomach; GB: gallbladder; CT: computed tomography.

A 61-year-old man with no known medical history presented with intractable nausea and vomiting for three days, after three weeks of recurrent heartburn, belching, early satiety, and poor appetite. Remote smoking history and rare use of alcohol were reported. On arrival, he was hemodynamically stable with an unremarkable physical exam. A non-contrast CT abdomen/pelvis revealed a markedly distended stomach without a clear obstructing lesion (Figure 1), with mild perigastric fat stranding around the gastric antrum and distal esophageal wall thickening. Preoperative laboratory values showed a mildly elevated total bilirubin and normal liver function enzymes (Table 1). Given the great clinical concern for GOO, an EGD was performed.

**Table 1:** Comparison of hepatic panel values before and after upper endoscopy ^a^Reference ranges may be affected by many variables, including the patient population and the laboratory methods used. The reference values used at Saint Peter’s University Hospital in New Brunswick are for adults who have no medical conditions that could affect the results.

Laboratory parameter	Preoperative value	Postoperative value	Reference range^a^	Units
Total bilirubin	1.3	0.4	0.1-1.2	mg/dL
Aspartate aminotransferase (AST)	39	23	17-59	U/L
Alanine aminotransferase (ALT)	17	19	0-50	U/L
Alkaline phosphatase (ALP)	58	48	56-119	U/L

**Figure 2:**
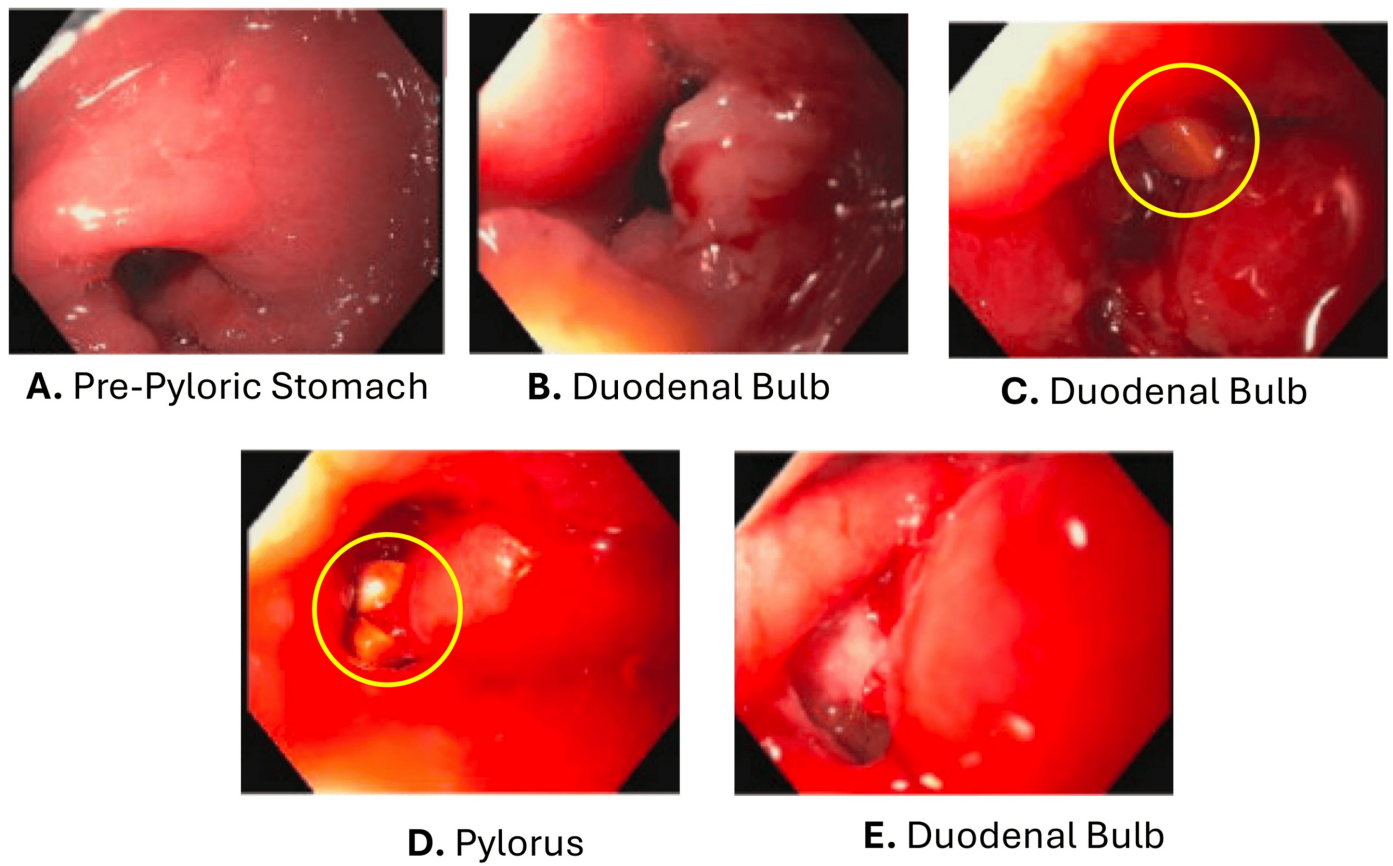
Upper endoscopy Endoscopic evaluation of the pre-pylorus, pylorus, and duodenal bulb; image A shows the pre-pyloric region of the stomach depicting nodular mucosa prior to punch biopsy; image B shows the severe stenosis of the duodenal bulb due to a medium-sized mass-like structure; images C and D depict the chelocystoduodenal fistulization tract and yellow stones within the fistula; image E shows the fistula tract after stone migration out of the tract during scope withdrawal.

Endoscopy showed nodular mucosa in the pre-pyloric region (Figure 2A) and a medium-sized infiltrative mass-like structure severely narrowing the first part of the duodenum (Figure 2B), which was biopsied. After traversing through the stenosis, the second portion of the duodenum appeared normal; however, gallstones immediately emerged, revealing a fistulous tract beneath the gallbladder (Figure 2C, 2D, 2E). Endoscopic stone extraction was not attempted due to the spontaneous calculi evacuation from the fistulous tract. The above findings were consistent for cholecystoduodenal fistula, raising concerns for duodenal or gallbladder malignancy. The stenosis was believed to contribute to the patient’s symptoms. Post-procedure, the patient showed clinical improvement, and surgery was consulted. A post-procedural evaluation of the hepatic panel showed normalization of the total bilirubin, and the liver function enzymes did not trend upward (Table 1). 

**Figure 3:**
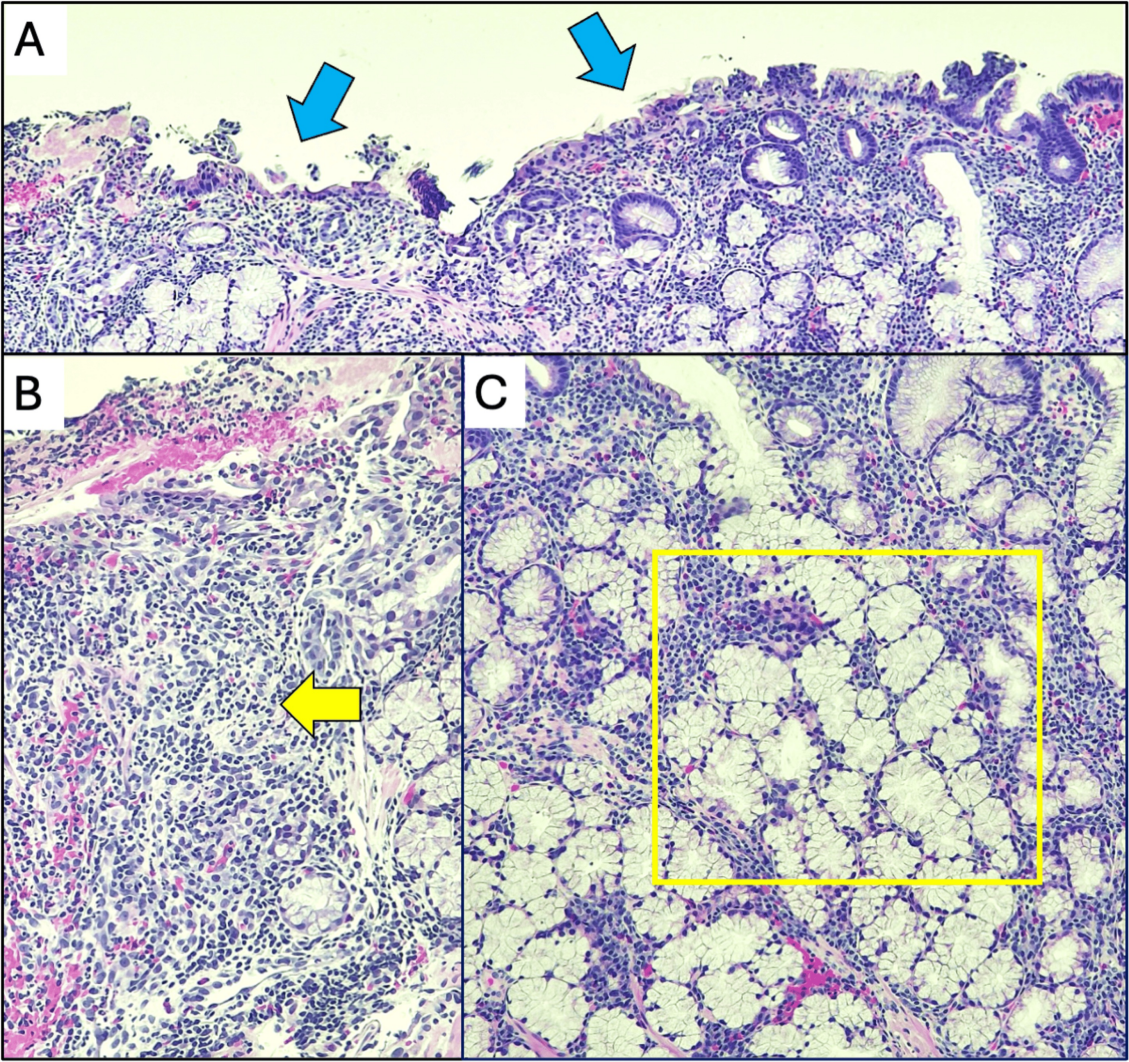
Hematoxylin and eosin staining of the duodenal mass-like structure A. Medium-power view showing duodenal mucosa with loss of normal villous architecture and ulceration (blue arrows). B. Medium-power view of the lamina propia showing signs of marked acute on chronic inflammation with dense neutrophilic infiltration (yellow arrow). C. Brunner's gland hyperplasia characterized by proliferation of lobulated mucus-secreting glands extending into the lamina propria (yellow frame).

**Figure 4:**
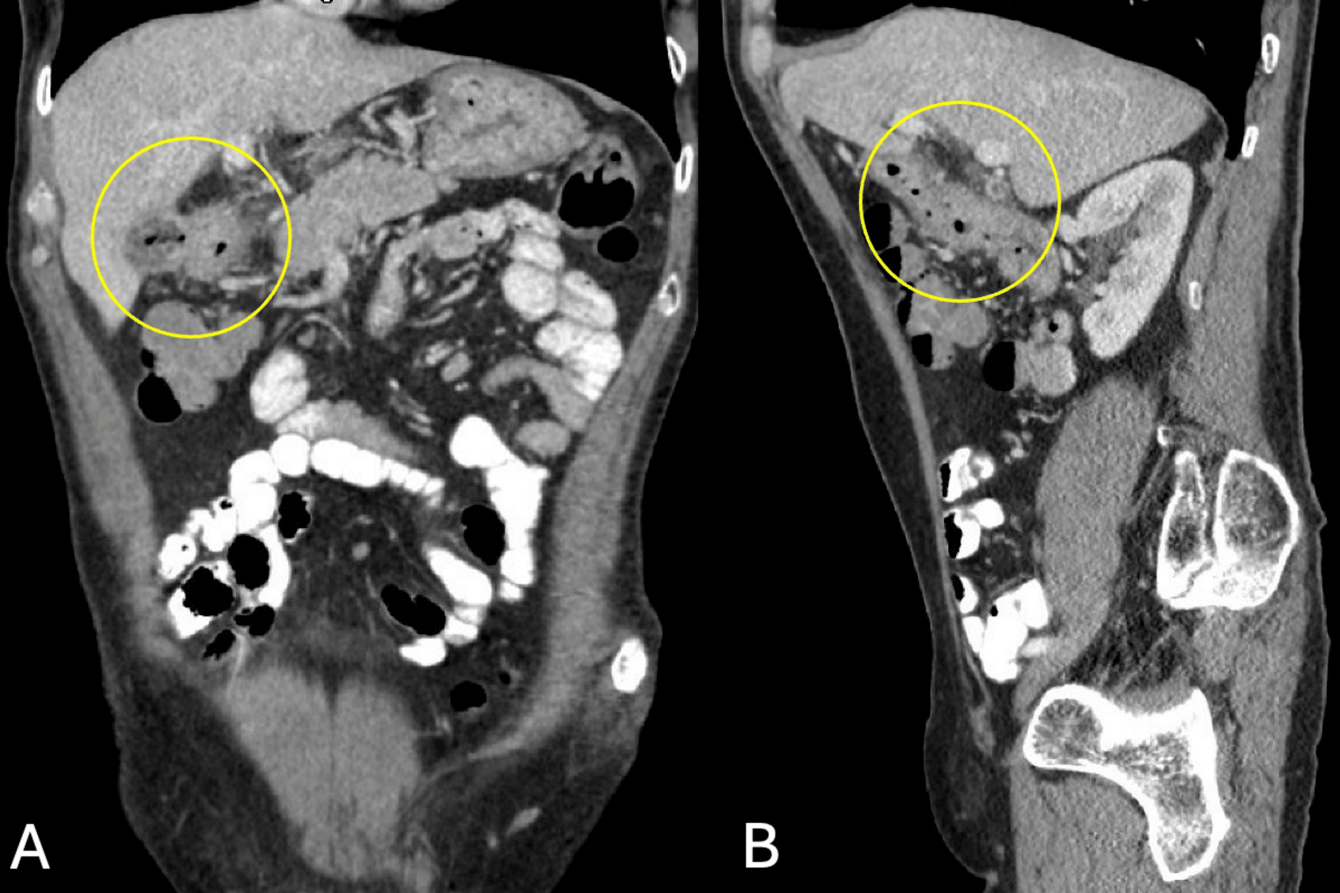
Contrast-enhanced computerized tomography of the abdomen and pelvis after upper endoscopy A. Coronal and B. sagittal views of the abdomen and pelvis with contrast depicting foci of air within the collapsed gallbladder and the contiguous gallbladder wall of the first part of the duodenum, with evidence of fistula extending from the duodenum into the gallbladder lumen. No evidence of air or dilation into the extra- and intrahepatic bile ducts.

Histopathological analysis of the biopsy specimen revealed duodenal mucosa with ulceration, marked acute and chronic inflammation, and Brunner’s gland hyperplasia (Figure 3). Immunostaining showed no malignancy. Tumor markers, including carcinoembryonic antigen (0.7 ng/mL) and cancer antigen 19-9 (3 U/mL), were within normal limits. Subsequent contrast-enhanced CT of the abdomen and pelvis revealed signs of cholecystoduodenal fistula, wall thickening of the first parts of the duodenum, and subcentimeter periduodenal and gastrohepatic ligament nodes (Figure 4). Based on the endoscopic findings and visualization of the fistula on imaging, the tract was estimated to be 5 mm in diameter and 55 mm in length from the duodenum to the gallbladder. The measurements were limited due to the patient's abnormal anatomy and contracted gallbladder. Eight weeks after discharge, the patient reported no symptoms and no further weight loss. The patient did not undergo a definitive surgical intervention due to personal reasons.

## Discussion

Bouveret syndrome is an uncommon form of gallstone ileus, which was first diagnosed preoperatively in the late 19th century by Leon August Bouveret [1]. It represents ~1-3% of cases, and is a rare cause of GOO, accounting for less than 1% of all GOO cases [6,7]. Bouveret syndrome results from the formation of a bilioenteric fistula, most commonly a cholecystoduodenal fistula, through which a large gallstone migrates and obstructs the duodenum or, less frequently, the stomach [8]. Typical patients are elderly women with chronic cholelithiasis [9], although our patient, a 61-year-old man, was atypical in terms of demographics.

The clinical presentation of this syndrome can be challenging due to its non-specific symptoms, delaying diagnosis and increasing complication risk [10]. Our patient had persistent nausea, intractable vomiting, and reflux symptoms, prompting an emergent CT scan, which revealed significant gastric distention and perigastric inflammation, an atypical presentation lacking the classic Rigler's triad. Neither oral nor intravenous contrast was administered initially, given the patient’s intolerance and to avoid unnecessary iodinated contrast exposure in an elderly patient. When imaging is limited, as in this case, endoscopy plays a critical role in establishing the diagnosis. In our patient, EGD revealed a duodenal mass-like stenotic region, along with a cholecystoduodenal fistula with posteriorly filled calculi. In line with the literature, cholecystoduodenal fistulas are exceedingly rare, with most cases involving a single cholecystoenteric tract rather than communication with both the stomach and duodenum [11]. Because fistula dimensions are infrequently and inconsistently reported in Bouveret syndrome, our case adds objective quantitative anatomic detail and contributes to an undercharacterized aspect of the condition. Measured fistula caliber is variable, commonly ~1 cm, and can be larger (2-3 cm) in some cases, but can also be only a few millimeters (Table 2) [12-16]. Notably, the fistula of our patient had a small-caliber, elongated morphology, which may still permit passage of a large calculus depending on the stone orientation and progressive dilation. Concomitant malignancy in the biliary tract in the setting of cholecystoduodenal fistula has been previously reported; however, no direct link is proven [17]. The biopsy of the mass-like stenotic region in our case did not show malignancy.

**Table 2:** Published reports and series documenting measured dimensions of biliary-enteric fistulas relevant to Bouveret syndrome, with emphasis on cholecystoduodenal fistulas This table summarizes selected case reports and a surgical case series that explicitly reported measured biliary-enteric fistula dimensions and the modality described in each source. CDF: cholecystoduodenal fistula: CGF: cholecystogastric fistula.

Study	Article type	Fistula type	Reported fistula dimension(s)	Measurement modality
Huang et al. (2022) [12]	Case series	CDF, CGF	1.4 cm in diameter	Intraoperative measurement
Nagata and Fujikawa (2024) [16]	Case report	CDF	3 cm in diameter	Intraoperative measurement
Vadioaloo et al. (2019) [13]	Case report	CDF	1 cm in diameter	Intraoperative measurement
Wang et al. (2019) [14]	Case report	CDF	2 cm in diameter	Intraoperative measurement
Hill et al. (2013) [15]	Case report	CDF	2 cm in length, 0.8 cm in diameter	Pathology report measurement

Management depends on patient factors and stone size or location. Endoscopic intervention is often the first-line approach, using nets or baskets, or lithotripsy, although the success rate varies with stone size and anatomy [18]. Some experts support that basket retrieval may work well for smaller stones and reduce surgical risk [7], but larger stones often require lithotripsy or surgery. Surgery is recommended when endoscopy fails or malignancy is suspected.

An ongoing challenge in Bouveret syndrome is fistula repair. If unrepaired, complications like recurrent gallstone ileus or pancreatitis may occur [19]. Some reports suggest increased gallbladder carcinoma risk in patients with cholecystoenteric fistulas, supporting cholecystectomy, especially in younger patients. Our patient improved clinically post-endoscopy, and surgery was consulted to consider definitive management, which was planned as an outpatient. Dietary modification, clinical monitoring, and endoscopic surveillance were offered to mitigate the risk of recurrence and potential complications.

## Conclusions

This case highlights the diagnostic complexity of Bouveret syndrome and underscores the need for a high index of suspicion in unexplained GOO. Despite its rarity, prompt endoscopic evaluation is crucial, especially when imaging is inconclusive. Endoscopic management can be effective and lead to preferable outcomes in individuals with small-sized calculi and for patients who cannot undergo surgery.
